# Pathophysiological Role of Vimentin Intermediate Filaments in Lung Diseases

**DOI:** 10.3389/fcell.2022.872759

**Published:** 2022-04-28

**Authors:** Ranu Surolia, Veena B. Antony

**Affiliations:** Division of Pulmonary, Allergy and Critical Care, Department of Medicine, University of Alabama at Birmingham, Birmingham, AL, United States

**Keywords:** vimentin (intermediate filaments), acute lung injury, chronic lung injury, host pathogen interactions, viral infections, bacterial infections, lung cancer, anti-vimentin antibodies

## Abstract

Vimentin intermediate filaments, a type III intermediate filament, are among the most widely studied IFs and are found abundantly in mesenchymal cells. Vimentin intermediate filaments localize primarily in the cytoplasm but can also be found on the cell surface and extracellular space. The cytoplasmic vimentin is well-recognized for its role in providing mechanical strength and regulating cell migration, adhesion, and division. The post-translationally modified forms of Vimentin intermediate filaments have several implications in host-pathogen interactions, cancers, and non-malignant lung diseases. This review will analyze the role of vimentin beyond just the epithelial to mesenchymal transition (EMT) marker highlighting its role as a regulator of host-pathogen interactions and signaling pathways for the pathophysiology of various lung diseases. In addition, we will also examine the clinically relevant anti-vimentin compounds and antibodies that could potentially interfere with the pathogenic role of Vimentin intermediate filaments in lung disease.

## Introduction

Vimentin, a type III intermediate filament, is one of the cell cytoskeleton proteins in mesenchymal cells (Herrmann et al., 1996) and is prominently associated with the maintenance of cell structure, and migration (Ivaska et al., 2007; Eriksson et al., 2009; Herrmann et al., 2009; Battaglia et al., 2018). The *Vim* ^*−/−*^ mice survive and grow normally (Colucci-Guyon et al., 1994). However, subsequent studies demonstrated that Vimentin intermediate filaments have crucial physiological roles in cell homeostasis (Gurland and Gundersen, 1995; Ivaska et al., 2007; Battaglia et al., 2018; Schaedel et al., 2021), and the *Vim* ^−/−^ mice and cells have altered functions under stress conditions (Henrion et al., 1997; Terzi et al., 1997; Eckes et al., 2000; Nieminen et al., 2006). These studies brought attention to the silent yet crucial role of Vimentin intermediate filaments in the pathophysiological arena. This review will focus on the role of Vimentin intermediate filaments in the pathogenesis of various lung diseases.

## Vimentin Intermediate Filaments and Post-Translational Modifications

Vimentin is a type III intermediate filament named by Frank and Weber in 1978 (Franke et al., 1978). The name vimentin was derived from the Latin word “vimentum,” which refers to arrays of flexible rods that can be arranged in both ordered (e.g., lattices, filigrees, and wicker-work) and non-ordered (e.g., brushwood) forms. As with other types III IFs, vimentin has a central *α*-helical “rod” domain flanked by the head (N-terminal) and tail (C-terminal) domains on both sides (Chernyatina et al., 2012). Vimentin intermediate filaments, as other intermediate filaments, must rearrange and reorganized during physiological and pathophysiological events that change the physical and functional properties of a cell. These processes are primarily driven by post-translational modifications (PTMs). PTMs of vimentin intermediate filaments change its shape, distribution, and interactions with other signaling molecules for the rapid modulation of its function under different conditions (Kraxner et al., 2021). The intrinsic polyelectrolyte nature of vimentin intermediate filaments (Janmey et al., 2014) is associated with non-enzymatic PTMs during redox imbalances (Perez-Sala et al., 2015; Wilson and Gonzalez-Billault, 2015)and can be mediated through enzymatic or non-enzymatic reactions. The interactions of Vimentin intermediate filaments with Ca^2+^ and Mg^2+^ increase assembly, crosslinking, and stiffness (Lin et al., 2010). Other non-enzymatic modifications are mostly oxidizing, resulting in glutathionylation, nitrosylation, or carbonylation of vimentin (West et al., 2006; Huang et al., 2009; Chavez et al., 2010; Griesser et al., 2021). The cysteine 328 (Cys 328/C328) is targeted by various oxidative modifications (Stamatakis et al., 2006; Gharbi et al., 2007; Perez-Sala et al., 2015). The cells transfected with GFP- tagged C328S vimentin (mutant) demonstrated presence of disassembled short squiggled and dots kind of GFP positive vimentin fragments (Perez-Sala et al., 2015). Hence, oxidation at C328 can cause disassembly of vimentin intermediate filaments.

The most common enzymatic PTM on vimentin intermediate filaments is phosphorylation (Sihag et al., 2007; Snider and Omary, 2014), and is essential for spatio-temporal regulation of its assembly, tissue-specific functions, and in some cases, diseases pathogenesis (Omary et al., 2006; Sihag et al., 2007; Snider and Omary, 2014; Snider et al., 2018). Multiple kinases, various chemical compounds, growth factors, cytokine treatments, viral infections can induce phosphorylation of vimentin intermediate filaments, and the details on these factors and phosphorylation sites is available at (https://www.phosphosite.org/proteinAction.action?id=2622&showAllSites=true). Specifically kinases such as protein kinase A (Inagaki et al., 1987), protein kinase C (Ivaska et al., 2005), cdc2 kinase (Chou et al., 1990; Chou et al., 1991; Chang et al., 2012), p21 activated kinase (Eriksson et al., 2004; Li et al., 2006), Rho-associated kinases (Goto et al., 1998; Sin et al., 1998), Akt1 (Zhu et al., 2011; Wang et al., 2012; Li et al., 2017b), Aurora-B (Goto et al., 2003), and CaMKIIA (Stefanovic et al., 2005) are well-known for phosphorylating vimentin.

PTMs other than oxidation and phosphorylation, include glycosylation (Snider et al., 2018; Tarbet et al., 2018), ubiquitination (Zhu et al., 2017; Cheng et al., 2019), sumoylation (Wang et al., 2010), acetylation (Guo et al., 2018) and citrullination (Inagaki et al., 1989). These PTMs on vimentin intermediate filaments are associated with but are not limited to stress sensing (Perez-Sala et al., 2015; Griesser et al., 2021), regulation of turnover of IF assembly (Herrmann et al., 2009), cell survival (Dinsdale et al., 2004), protein-protein interactions (Wang et al., 2012), and interaction with the nuclear membrane (Neelam et al., 2015). Sumoylation of vimentin by Protein Inhibitor of Activated STAT3 (PIAS3) inhibits glioma cell migration (Wang et al., 2010) while acetylation of vimentin intermediate filaments at K120 b SIRT5 increases metastasis in hepatocellular carcinoma (Guo et al., 2018). Citrullination of vimentin intermediate filaments leads to secretion of citrullinated vimentin (Cit-Vim) as an autoantigen implicated in the pathogenesis of rheumatoid arthritis (RA) (Vossenaar et al., 2004). Cit-Vim interacts with B cells to result autoimmunity in RA (Vossenaar et al., 2004; Valesini et al., 2015). Interestingly, the immunogenic properties of Cit-Vim peptides are being explored to develop an anti-cancer vaccine (Brentville et al., 2020). In recent studies, the pathological role of Cit-Vim in chronic lung diseases like COPD, pulmonary fibrosis, and sarcoidosis have been explored (Vassallo et al., 2014; Lugli et al., 2015; Ytterberg et al., 2015; Musaelyan et al., 2018; Nissen et al., 2019; Li et al., 2021).

## Vimentin Intermediate Filaments in Pathological Roles in Lung Diseases

With the widespread use of specific antibodies, high-resolution microscopy techniques, and other advanced techniques, it has become evident that the differential amount and forms of vimentin and auto-antibodies to vimentin are present in the bronchoalveolar lavages, cells, and lung tissues from patients with various lung diseases demonstrating the pivotal role of vimentin in their pathogenesis (Rho et al., 2009; Wahlstrom et al., 2009; Li et al., 2017a; Musaelyan et al., 2018; Surolia et al., 2019; Zaccardelli et al., 2019; Li et al., 2021; Zaccardelli et al., 2021). These multiple forms of Vimentin intermediate filaments are comprehensively shown to be involved in inflammation (Benes et al., 2006; Dos Santos et al., 2015; Lam et al., 2018; Yu et al., 2018; Lam et al., 2020), and host-pathogen interactions (Garg et al., 2006; Babrak et al., 2015; Mahesh et al., 2016; Yu et al., 2016; Zhang et al., 2020) in non-malignant acute lung injuries (trauma, viral infections, bacterial infections, etc.) and chronic lung diseases (IPF, autoimmune ILDs, COPD, and asthma) (Li et al., 2017a; Musaelyan et al., 2018; Nissen et al., 2019; Surolia et al., 2019; Li et al., 2021). Additionally, vimentin is a gold-standard marker of epithelial–to-mesenchymal differentiation during malignancies (Satelli and Li, 2011; Bogush et al., 2020), and is also proposed as a diagnostic and prognostic marker in lung cancers (Rho et al., 2009; Dauphin et al., 2013; Rodriguez et al., 2013; Havel et al., 2015; Teocharoen et al., 2021).

### Acute Lung Injury/Acute Respiratory Distress Syndrome

**FIGURE 1:**
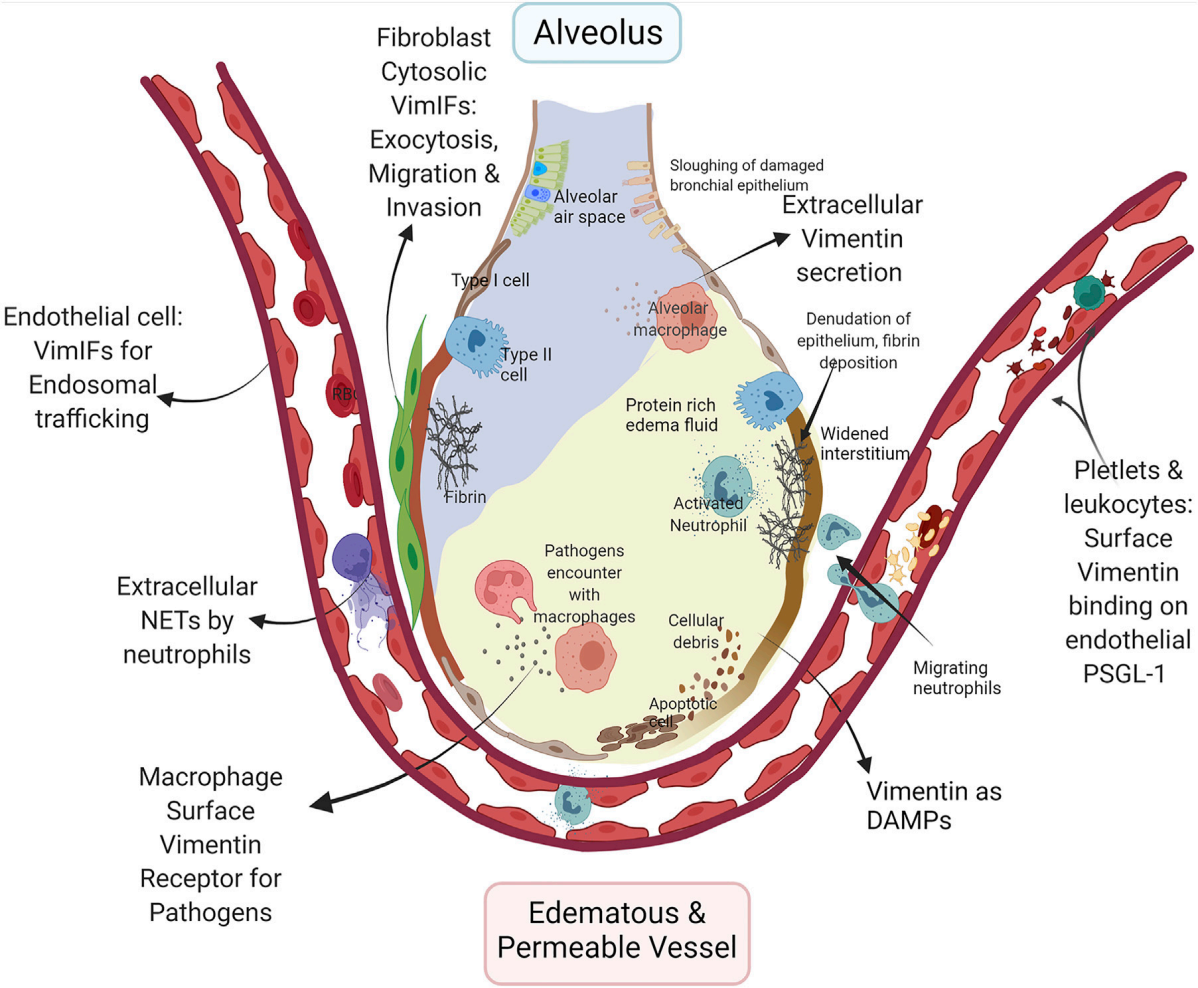
Multifaceted role of vimentin in ARDS/ALI: Increased permeability of the alveolar-capillary membrane and pro-inflammatory conditions are fundamental characteristics of ALI. The schematic demonstrate very few epithelial cells due to increased cell death, and the intra-alveolar space depicted represents the alveolar lining fluid that is in contact with the air. The increased expression of surface vimentin on endothelial cells enhances lymphocyte adhesion and transmigration across endothelial cells through PSGL-1 binding on the lymphocytes, including neutrophils. Expression of vimentin and dynamics of Vimentin intermediate filaments regulate the endosomal signaling through Rab GTPase to transport VE-cadherin to the cell surface in endothelial cells for the maintenance of barrier functions. The PTM on vimentin in neutrophils leads to neutrophil extracellular traps (NETs) *via* netosis. NETs disrupt the microvascular endothelial barrier, increasing edematous and permeable vessels and causing a protein-rich fluid influx in the airspace. The increased expression of vimentin on platelets increases vitronectin and PAI-1 complex formation, which may provide stabilization of thrombi. The fibrins and fibrinolysis-related enzymes cause the dissolution of epithelial surface proteins and denudation of the epithelial barrier layer. Vimentin expression in fibroblasts regulates exocytosis and invasion, contributing to their proliferation in ALI. The surface vimentin on macrophage is essential for several bacteria and viruses for the host cell invasion and phagocytosis. Secreted vimentin from dead cell debris and activated immune cells act as DAMPs. The figure was created with BioRender.com.

ALI is a broad term encompassing the pathophysiology of diffuse alveolar injury by toxin inhalation or as a consequence of systemic diseases, such as sepsis, severe shock, and trauma (Johnson and Matthay, 2010). The activated lymphocytes, cytokines, and Damage-Associated Molecular Patterns (DAMPs) weave a redundant inflammatory network for the development and progression of ALI (Tolle and Standiford, 2013). Various forms of vimentin regulate lymphocyte differentiation, activation, and inflammation through inflammasomes and act as DAMPs, signifying its multipronged role in the development of ALI (Dellagi et al., 1983; Mor-Vaknin et al., 2003; Benes et al., 2006; Nieminen et al., 2006; Dos Santos et al., 2015; Lam et al., 2018; Yu et al., 2018; Su L. et al., 2019; Su L.-X. et al., 2019; Lam et al., 2020) (Figure 1).

The differentiation of v-myb-transformed BM2 monoblasts cells to macrophage-like cells is dependent on the expression of vimentin (Benes et al., 2006). The migration and extravasation of monocytes through endothelial cells rely on vimentin intermediate filaments in inflammatory conditions (Nieminen et al., 2006; Lam et al., 2018). Furthermore, anti-vimentin antibodies decrease ROS generation in macrophages, inferring that the surface vimentin is pro-inflammatory (Mor-Vaknin et al., 2003) and are essential for killing bacteria and other pathogens (Forman and Torres, 2001). In addition to improving the anti-bacterial function of macrophages, a recent study demonstrated that the extracellular vimentin modulates the activity of LPS- activated dendritic cells and reduces Th1 differentiation (Yu et al., 2018).

Sepsis is an extreme immune response to an infection, where overactivation of innate immune response and immunosuppression are responsible for complex immunopathology that causes ARDS. The apoptosis of lymphoid cells after the acute phase sepsis and suppression of lymphoid cell activity contribute to infection-related complications, seen in septic shock (Delano and Ward, 2016) (Su L.-X. et al., 2019). The suppression of vimentin in LPS treated macrophages showed increased inflammatory mediator, TNF-*α*. In contrast, it decreases the anti-inflammatory cytokine IL-10. Patients with sepsis and septic shock have increased levels of vimentin in serum. The disruption of Vimentin intermediate filaments in lymphocytes results in increased cell death and the release of soluble vimentin into blood circulation, which is related to the worse outcome of sepsis (Su L. et al., 2019). These regulatory responses of vimentin in the LPS injury model demonstrate the role of Vimentin intermediate filaments in immunosuppression during sepsis.

Recombinant extracellular vimentin has been shown to inhibit the infiltration of neutrophils into the lungs of the LPS-ALI mouse model (Lam et al., 2018). Extracellular vimentin itself acts as DAMP (Yu et al., 2018). In a pro-inflammatory environment, vimentin can be secreted by macrophages, monocytes (Mor-Vaknin et al., 2003), neutrophils (Kaplan, 2013), endothelial cells (Li et al., 2017a), apoptotic lymphocytes (Boilard et al., 2003), apoptotic neutrophils (Moisan and Girard, 2006), and injured skeletal muscle cells (Bryant et al., 2006) can secrete extracellular vimentin due to overexpression, traumatic cell injury, or cell death (Mellgren, 2010). As a DAMP, extracellular vimentin suppresses the pro-inflammatory adaptive immune responses by blocking the secretion of pro-inflammatory cytokines IL-12 and IL-6 from LPS stimulated dendritic cells (Yu et al., 2018).

The direct role of Vimentin intermediate filaments in innate immunity was demonstrated in a groundbreaking study showing that the inflammasome activation has obligatory requirements of interaction with vimentin (Dos Santos et al., 2015). Inflammasomes are molecular complexes comprised of basic protein units, including receptors and sensors that regulate the activation of caspase-1 and IL-1*β* (Guo et al., 2015). In macrophages, vimentin regulates innate immunity by regulating NACHT, LRR, and PYD domains-containing protein 3 (NLRP3) inflammasome pathway (Dos Santos et al., 2015). The inflammasome is a complex made of NLRP3, ASC (apoptosis-associated speck-like protein containing a CARD), and caspase-1. Vimentin intermediate filaments act as scaffolds to form this complex. The interaction of NLRP3 with Vimentin intermediate filaments occurs *via* macrophage inhibitor factor (MIF) (Lang et al., 2018), which activates inflammasomes. This study demonstrated that the vimentin-deficient mice exhibit attenuated ALI after the lipopolysaccharide (LPS) challenge, as represented by reductions in inflammation, IL-1*β* levels, and endothelial permeability (Dos Santos et al., 2015). Inflammasome complexes and their downstream products are involved in viral infections, bacterial infections, COPD, asthma, and ARDS, which have been reviewed in depth elsewhere (Dos Santos et al., 2012; Howrylak and Nakahira, 2017; Liu et al., 2021; Vora et al., 2021).

Acute lung injuries are associated with neutrophilia, alveolar-capillary membrane destruction, and increased permeability (Figure 1), mechanisms of which have been examined in detail (Lin and Fessler, 2021). The exaggerated extravasation and migration of leukocytes through pulmonary blood capillaries are dependent on P-selectins. P-selectin glycoprotein ligand-1 (PSGL-1) on leukocytes binds to P-selectin on platelets and endothelium wherein vimentin can act as an endogenous ligand for P-selectin. The treatment with recombinant vimentin attenuates ALI, plausibly by occupying P-selectin on endothelium which makes it is unavailable for the binding to PSGL-1 of leukocytes and platelets (Lam et al., 2018; Lam et al., 2020). Moreover, Vimentin intermediate filaments indirectly affect neutrophil-mediated ALI by regulating non-apoptotic neutrophil cell death, known as netosis (Brinkmann et al., 2004). During netosis, cellular chromatin is expelled out of the neutrophil, and the expelled chromatins are called neutrophil extracellular traps (NETs) that are decorated with granular proteins and proteases of neutrophils (Papayannopoulos et al., 2010), and these NETs are responsible for increased permeability of microvascular endothelium leading to ALI (Surolia et al., 2021). The NETs themselves can trigger NLRP3 inflammasomes for a sterile inflammation (Allam et al., 2013). The process of netosis is dependent on the citrullination of vimentin intermediate filaments, which leads to their disassembly. The disassembled of vimentin intermediate filaments is essential for the rounding of nucleus in neutrophils, and initiation of decondition of chromatin for netosis (Thiam et al., 2020).

Pulmonary edema in ARDS results from increased microvascular permeability. Vimentin intermediate filaments can indirectly regulate permeability through their functions as endosomal trafficking regulators. Vimentin intermediate filaments interact with endocytosis regulator proteins, namely Rab GTPase family proteins (Cogli et al., 2013; Margiotta et al., 2017; Romano et al., 2021). During edema, the inter-endothelial junctions are maintained by vascular endothelial cadherins (VE-cadherin). Recently, a study demonstrated Rab GTPases, Rab4, -7, and -9 regulate vascular permeability through enhanced VE-cadherin expression at the interendothelial junction (Chichger et al., 2016). Rab7a and Rab9 interactions with vimentin are indispensable for efficient endosome trafficking (Cogli et al., 2013; Margiotta et al., 2017; Romano et al., 2021). It is reasonable to assume that the VE-cadherin exosomes require Rab7 and Rab9 interactions with vimentin intermediate filaments for their successful shuttling to the surface of endothelial cells. Any modulation in the dynamics of PTMs of vimentin intermediate filaments can hamper this endothelial endosomal trafficking to cause edema in ALI.

ARDS/ALI patients have imbalances in coagulation and fibrinolysis pathways, which causes the increased presence of fibrin-rich exudates in the lumen of lung alveoli. Platelets aggregate complexes with fibrin to form stabilized clots in ARDS. Increased expression of vimentin on the surface of platelets polymerizes vitronectin to form a complex with the active form of plasminogen activator inhibitor-1 (PAI-1) (Podor et al., 2002), which stabilizes the thrombus (Konstantinides et al., 2001). This increased fibrin deposition increases ALI permeability by myriads of pathways (Bastarache, 2009). Additionally, the formation of micro thrombi is also a common coagulation related pathology of ARDS that affects the microvascular endothelium. Vimentin intermediate filaments may have an indirect role in the increased micro thrombosis *via* the regulation of exocytosis (Faigle et al., 2000). *Exocytosis* is a normal process that releases the cell contents to the cell’s exterior (Sollner, 2003). The exocytosis of abnormal VWF by endothelial cells causes micothrombosis in ARDS. During microthrmbosis, endothelial cells exocytose von Willebrand Factor (VWF), forming microthrombi complexes with activated platelets.

Moreover, exocytosis is a prerequisite for the migration and invasion of fibroblasts (Bretscher, 2008). Vimentin intermediate filaments act as a reservoir for a vesicle docking and fusion protein regulator, SNAP23 (Faigle et al., 2000). Vimentin intermediate filaments associated with reservoirs has been shown to traffic SNAP23 from the available plasma membrane pool (Faigle et al., 2000). Any PTM or disruption in vimentin intermediate filaments may modulate its availability to form SNARE complexes for exocytosis Specifically, Vimentin intermediate filaments regulated exocytosis may be necessary for the increased migration of fibroblasts and their invasion into fibrinous exudate alveolar spaces (Quesnel et al., 2010). It is not surprising that the BALs from the patient with ALI demonstrate the presence of alveolar fibroblasts, with increased expression of vimentin that is of a persistently activated phenotype with enhanced collagen- 1 producing and migratory capacity (Quesnel et al., 2010). Moreover, FGFs released from fibroblasts attenuate acute lung injury in the LPS model of ALI (Tong et al., 2016). More investigations are required to explore the direct role of fibroblasts in ALI.

### Respiratory Viral Infections

Mounting evidence demonstrates the vital role of vimentin intermediate filaments and their soluble forms in virus-host cell interactions (Ramos et al., 2020; Zhang et al., 2020). Vimentin intermediate filaments affect infection, virulence, and replication of viruses in the host cells. For some viral infections, expression of Vimentin intermediate filaments on the cell surface aid at an early stage of infection as a co-receptor for the entry into the host cell (Thomas et al., 1996; Kim et al., 2006; Das et al., 2011; Du et al., 2014). For example, the human immunodeficiency virus (HIV) infects the host cell by making a pre-integration complex with vimentin present on the cell surface. The V3 region of HIV-1 and host surface vimentin interact to form the pre-integration complex after viral binding on the host CD4 receptor. After forming a pre-integration complex, the proteases from HIV-1 cleave vimentin intermediate filaments leading to its collapse towards the nuclear pore, thus bringing the virus into the nuclear entry site (Thomas et al., 1996). Vimentin intermediate filaments can modulate the replication, assembly, and egress of viruses in the host due to their known function of regulating endosomal trafficking *via* Rab7a and Polo-like kinase 1 (Plk1). Rab7a, which is ubiquitously present in early and late endosomes (Aloisi and Bucci, 2013; Guerra and Bucci, 2016), interacts with the insoluble and soluble vimentin (Cogli et al., 2013; Margiotta et al., 2017). Rab7a interacts directly with vimentin, and this interaction modulates vimentin phosphorylation and assembly (Cogli et al., 2013). Rab7a depleted cells have an abundance of insoluble Vimentin intermediate filaments, and defective endosomal trafficking (Romano et al., 2021). Phosphorylation of Vimentin intermediate filaments at Ser459 by Polo-like kinase 1 (Plk1) inhibits the endolytic fusion during mitosis (Ikawa et al., 2014). Altogether these interactions demonstrate vimentin as a critical regulator of late endocytic trafficking and egress of viral particles (Risco et al., 2002; Fay and Pante, 2013; Wu and Pante, 2016; Sabharwal et al., 2019). In another strategy, African swine fever virus, Vaccinia virus, and Enterovirus trigger rearrangement of Vimentin intermediate filaments as cages around the viral replication factories (Risco et al., 2002). These viruses utilize Vimentin intermediate filaments cages to egress and incorporate viral proteins and DNA for its replication (Stefanovic et al., 2005; Turkki et al., 2020). A more comprehensive elaboration on the role of vimentin during host-virus interactions in a wide range of viral infections is described elsewhere (Ramos et al., 2020; Zhang et al., 2020).

**FIGURE 2:**
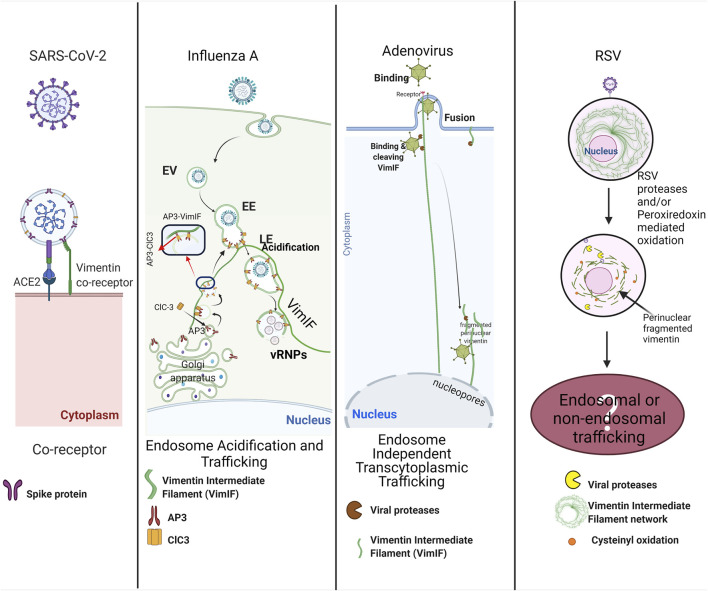
Multiple roles of vimentin in viral respiratory infections: SARS-CoV2 exploits surface vimentin co-receptor for the entry in the host cell. Influenza A virus utilizes vimentin regulated trafficking of late endosomes for the release of vRNPs near the host nucleus. Adenoviruses bind to vimentin intermediate filaments (VimIFs) after host cell invasion, and the viral proteases cleave vimentin intermediate filaments to shuttle viruses in the vicinity of the host nucleus, also called endosome independent transcytoplasmic trafficking. RSV infection causes cleaving of VimIFs and accumulation of VimIF in the peri-nuclear region. Alterations in VimIFs may be associated with endosomal trafficking or non-endosomal trafficking for the shuttling virus next to the nucleus for its replication. The figure was created with BioRender.com.

Unfortunately, respiratory tract viral infections are a leading cause of morbidity and mortality, where the symptoms can range from mild or asymptomatic upper airway infections to severe pneumonia. The most common respiratory viruses are SARS-CoV-2, influenza, respiratory syncytial virus (RSV), and adenoviruses (Figure 2).

#### Coronaviruses

A coronavirus classified as a member of the Coronaviridae family was identified as SARS-CoV-1 as the causative pathogen of the severe acute respiratory syndrome (SARS) in 2002. Since then, MERS-CoV and SARS-CoV-2 have been identified to cause severe illnesses in humans, such as the Middle East respiratory syndrome (MERS) and COVID-19. Currently, SARS-CoV-2 has precipitated a global public health crisis of our times with more than 25 million infected people up to date worldwide (Nov. 2021), and still continues unabated. The transmembrane spike (S) glycoprotein of SARS-CoV-2 and SARS-CoV have similar affinities to bind on human angiotensin-converting enzyme 2 (ACE2) (Walls et al., 2020). Interestingly, cell surface vimentin is identified as a co-receptor for binding SARS-CoV spike proteins (Yu et al., 2016) and it also acts as a co-receptor for SARS-CoV-2 spike proteins (Suprewicz et al., 2021; Thalla et al., 2021; Amraei et al., 2022). Recent study demonstrated that the coexpression of vimentin with ACE2 increased SARS-CoV-2 entry in HEK-293 cells, and the inhibition of vimentin expression decreased the SARS-CoV-2 infection of human endothelial cells (Amraei et al., 2022). Treatment with anti-vimentin antibodies considerably decreased the virus infection, which shows the direct role of surface vimentin in the binding of virus spike proteins (Yu et al., 2016). These reports do not clarify which domain of vimentin interacts with viral spike protein. Enterovirus 71 (EP71) and Cowpea mosaic virus (CPMV) utilized the tail region of vimentin as a receptor for the entry in host cells (Koudelka et al., 2009; Du et al., 2014); we postulate that the tail region of vimentin interacts with the SARS-CoV spike proteins. Overall, vimentin has a role in binding to SARS Co-V and SARS-CoV2 virus (Figure 2). Nevertheless, more in depth studies are warranted to consolidate these findings.

#### Influenza

Influenza A and B infections, commonly known as flu, cause contagious respiratory tract illness by causing upper respiratory tract infections (URTI) and sometimes lower respiratory tract infections (LTRI). An early study showed that disruption of Vimentin intermediate filaments impairs virus production (Arcangeletti et al., 1997), whereas proteomic data support the interaction of vimentin with viral ribonucleoprotein complexes (vRNPs) (Mayer et al., 2007). Later, a detailed study in *vim* ^*−/−*^ cells demonstrated that vimentin regulates endosomal trafficking to release vRNPs in the cytoplasm from the late endosomes, maintaining pH in the endosomes (Wu and Pante, 2016). The regulation of acidification of endosomes can be attributed to the sorting the endosomal chloride channel-3 (ClC-3), a chloride channel and transporter responsible for the endosomal acidification (Hara-Chikuma et al., 2005). The sorting of ClC-3 into the synaptic vesicles is managed by adapter protein-3 (AP-3) (Salazar et al., 2004). Adaptor proteins are protein-binding modules that link protein-binding partners together and facilitate the creation of larger signaling complexes. AP-3 is an essential adapter protein for lyso-endosomal sorting machinery (Odorizzi et al., 1998) that interacts with vimentin intermediate filaments for the sorting of proteins for endosomes formation and their trafficking (Styers et al., 2004). Based on these studies, it is established that vim^−/−^ cells demonstrate decreased acidification of endosomes due to a loss of Vimentin intermediate filaments-AP-3 interactions that would have a negative effect on the sorting and distribution of ClC-3 in the late endosomes (LE). Hence, the decrease in the number and virulence of virions released by vim^−/−^cells (Wu and Pante, 2016) can be attributed to the decreased endosomal acidification as discussed (Figure 2).

#### Adenoviruses

Adenoviruses are DNA viruses that typically cause mild infections involving the upper or lower respiratory tract (Berk, 1991; Lynch and Kajon, 2016). Studies have shown that the adenoviral serotypes requiring endosome independent trans-cytoplasmic penetration routes have proteases that cleave vimentin intermediate filaments (Belin and Boulanger, 1987; Defer et al., 1990) (Figure 2). Although the function of cleaved vimentin is not described in these studies, it is possible that cleaved vimentin could transport the adenovirus directly to the perinuclear region due to collapse of vimentin intermediate filament network similar to HIV-1 infections (Thomas et al., 1996).

#### Respiratory Syncytial Virus

RSV infects airway mucosa to cause uncomplicated upper respiratory tract infections but can also spread to the lower respiratory tract and are mainly associated with bronchiolitis that can be deadly in children younger than 5 years of age (Shi et al., 2017). Although the direct role of vimentin intermediate filaments in the infection and the life cycle of RSV is not yet explored, RSV infections modulate the activity and expression of host superoxide dismutase (SOD) 1, 2, and 3; catalase, glutathione peroxidase (GPx), and glutathione S-transferase (GST) that leads to increased auto-oxidation of proteins in the cell (Hosakote et al., 2009). It is reported that RSV induces cysteinyl oxidation and decreases the expression of vimentin (Garcia-Barreno et al., 1988; Jamaluddin et al., 2010). Cysteinyl oxidation is an example of oxidative stress-mediated disruption of the vimentin intermediate filaments network and may have pathophysiological implications (Monico et al., 2019). In another study, the RSV mediated modulations in peroxiredoxins 1 and, 4 (Prdx-1 and Prdx-4) were shown to be responsible for the oxidation of nuclear intermediate filament complexes, including vimentin (Jamaluddin et al., 2010). The oxidation of Vimentin intermediate filaments may disturb the nuclear mechanical homeostasis (Neelam et al., 2015) in infected cells, but further studies are required to evaluate the specific role of oxidized Vimentin intermediate filaments in RSV infections (Figure 2).

In addition to virus-host interactions and viral life cycle, Vimentin intermediate filaments are associated with the pathogenesis of viral infections mediated ALI. Studies have demonstrated that RSV-induced netosis has a significant role in lung injury (Muraro et al., 2018; Mutua et al., 2021). In the above ALI section, we have discussed the possible role of citrullination of Vimentin intermediate filaments as an initiating step for the decondensation of chromatin and rupturing the nucleus during netosis (Thiam et al., 2020). Hence, Vimentin intermediate filaments have an indirect role in promoting RSV infection mediated acute lung injury.

Of note, the versatile forms and different localization of Vimentin intermediate filaments play a critical role in various stages of viral life cycles and following inflammatory pathways during viral lung infections. Targeting specific forms of vimentin can be utilized as one of the multiple strategies to inhibit viral entry in the host cell.

### Respiratory Bacterial Infections

Bacterial infections are severe and prevalent among immunocompromised people (Al-Saad et al., 2008; Ahmed et al., 2011). Macrophages are the first line of defense to phagocytize and kill bacteria (Allard et al., 2018). The host-pathogen interaction mediated by macrophages and lymphocytes is crucial, and any discrepancy leads to serious bacterial infections in the lung and the development of pneumonia and pleurisy (Al-Saad et al., 2008). This section will describe the role of Vimentin intermediate filaments in the host-pathogen interactions and concomitant development of pathological features.

*Mycobacterium tuberculosis* (*M.tb.*) infections and non-tuberculosis *mycobacterium* infections are common forms of bacterial infections of the lungs in many parts of the world. During *M.tb.* infections, natural killer (NK) cells kill autologously-infected cells without prior sensitization as an innate immune response (Perera Molligoda Arachchige, 2021). The monocytes infected with *M. tb.* H37Ra have upregulated surface expression of vimentin compared to the uninfected monocytes. The NK cells recognize these infected cells by binding the NKp46 ligand to vimentin expressed on *M.tb.* H37Ra infected monocytes (Garg et al., 2006). Furthermore, the same study demonstrated that the neutralization of vimentin reduces the capacity of NK cells to lyse *M.tb.* H37Ra -infected alveolar macrophages. In another study, PKA/PKC mediated phosphorylation of vimentin was demonstrated to differentiate monocyte to macrophage, and these newly differentiated macrophages showed downregulation of expression of vimentin after infection with live *M.tb.* H37Rv infection *via* an ESAT-6 dependent mechanism (Mahesh et al., 2016). The apparent discrepancy of these results can be explained by the differences in the virulence of the mycobacterial strain used for the studies. *M.tb.* H37Ra is an attenuated *Mycobacterium* strain, whereas the *M.tb.* H37Rv is a virulent strain. *M.tb.* H37Ra exhibits significant alterations to either the genome or the expression of virulence genes compared to the virulent variant *M.tb.* H37Rv (Brosch et al., 1999; Li A. H. et al., 2010). The differential response for vimentin expression by these strains points towards the importance of surface vimentin expression in the host immune cell interactions and innate immunity. Virulent *mycobacterium* infection may inhibit the lysis of infected macrophage by NK cells by downregulating vimentin expression. Several mechanisms are altered by virulent strains of *mycobacteria* for the prolonged survival in infected macrophages to increase the intracellular bacterial burden inside the infected macrophages.

The instrumental role of surface vimentin in the host cell invasion has also been demonstrated in infection by *M. avium subsp. Hominissuis*. In order to achieve efficient mucosal invasion, *M. avium* forms microaggregates on the surface of the host cells, facilitating bacterial microaggragate binding protein 1 (MBP-1) by binding and polymerizing with the host cell surface vimentin.

The interaction of MBP-1 and host cells surface vimentin was shown to be inhibited by anti-vimentin antibody treatment in HEp-2 cells, suggesting that polymerized vimentin expression is vital for *M. avium* adherence to the host cell (Babrak et al., 2015). In addition to host-pathogen interactions, vimentin may also affect the subsequent pathological features of infection in the lung, such as granuloma formation. Granulomas are a compact and organized structure formed by the initial aggregation of infected macrophages and are a salient feature of *tuberculosis* and non-tuberculosis mycobacterial infections (Rubin, 2009). We and others have shown that the necrotic cell death of the infected granulomatous macrophages is associated with the dissipation of the bacteria by breaking the compact structure of granulomas (Russell et al., 2009; Regev et al., 2012; Silva-Gomes et al., 2013; Surolia et al., 2016). The breakdown of granuloma due to necrotic core dissipates bacteria dysregulates the immune response leading to lung tissue destruction and morbidity. Interestingly, the tight and well-formed granuloma are found to be rich in vimentin on their periphery (Kaarteenaho-Wiik et al., 2007). The direct role of vimentin is not understood in these structures and can be related to increased fibroblastic scar formation around the infected macrophages and lymphocytes aggregates (Kaarteenaho-Wiik et al., 2007). Recently, computational experimentation and wet-lab experimental approaches demonstrated the possibility of transforming vimentin-rich macrophages, which can differentiate into the myofibroblasts like cells around the macrophage aggregates in the later stages of granuloma formation (Evans et al., 2020).

*Sarcoidosis* is an idiopathic lung disease that features granuloma formation (Heinle and Chang, 2014). There is no clinical observation-based evidence for intracellular pathogen inside the sarcoidosis granuloma, yet few studies have demonstrated the plausible presence of dormant *mycobacterium* (Esteves et al., 2016) in vimentin-positive antigen-presenting cells (Wahlstrom et al., 2007; Chen et al., 2008; Wahlstrom et al., 2009). The discovery of the presence of residual *mycobacterium* antigens such as catalase-peroxidase (mKatG), superoxide dismutase A (Sod A), ESAT6, and *M. tuberculosis* heat shock proteins (Mtb-HSP) in the granulomatous lymphocytes roots to the hypothesis for the presence of a dormant form of *mycobacterium*. Vimentin intermediate filaments are well-recognized auto-antigens in sarcoidosis (Kinloch et al., 2018) and are shown to cause clonal expansion of lung-specific V*α*2.3 + V*β*22 + CD4 + T lymphocytes in the granuloma (Kinloch et al., 2018). These observations suggest that the presence of surface vimentin on the host cells may be involved in granuloma formation, and future studies are warranted in this understudied area. Overall, these scattered observations namely, the differential expression of vimentin in infected cells, polymerization of vimentin on the cell surface, presence of vimentin as antigen in granuloma presenting lymphocytes, and presence of Vimentin intermediate filaments rich cells in the peripheral fibroblastic case around aggregated lymphocytes, may have an inter-dependent or independent role of vimentin in the granuloma formation and progression of the disease.

### Chronic Lung Diseases

Owing to its importance as a mesenchymal marker, the expression of vimentin is extensively demonstrated during lung remodeling as one of the driver for the pathogenesis of chronic lung diseases (Kage and Borok, 2012; Rout-Pitt et al., 2018). Different PTMs on Vimentin intermediate filaments have been explored for their regulatory role in development of chronic lung diseases. The post-translational modification of Vimentin intermediate filaments such as citrullination, carbamylation, and phosphorylation is associated with the pathogenesis of chronic lung diseases namely, idiopathic pulmonary fibrosis (IPF) (Li et al., 2017a; Li et al., 2021), chronic obstructive pulmonary disease (COPD) (Lugli et al., 2015; Nissen et al., 2019), rheumatoid arthritis-associated interstitial lung disease (RA-ILD) (Lugli et al., 2015), and asthma (Zaccardelli et al., 2019; Zaccardelli et al., 2021).

#### Role of Vimentin in Lung Fibrosis

Interstitial lung diseases (ILD) refer to a collection of disorders characterized by varying degrees of inflammation and fibrosis in the lung interstitium. The most common form of idiopathic ILD is IPF. The firsthand evidence of extracellular and autoimmune forms of vimentin in IPF came from our study showing the presence of anti-vimentin antibodies that were associated with the worse clinical outcomes in the patients with IPF (Li et al., 2017a). We demonstrated that the binding of this anti-vimentin antibodies on HLA-DR was associated with the proliferation of CD4 T cells and enhanced IL-4, IL-17, and TGF-*β*1 levels (Li et al., 2017a). The transplant-free survival was higher in the patients with lower anti-vimentin autoantibodies. Furthermore, our study also demonstrated that environmental cadmium (Cd) exposures and smoking increased citrullinated vimentin in the bronchoalveolar lavages and serum of patients with IPF (Li et al., 2021) suggesting that citrullinated vimentin acts as a spearhead of inflammatory reactions that over time give rise to fibrotic scar formation of the lung and cause IPF. The peptidyl arginine deiminase 2 (PAD2) mediated citrullination of vimentin solubilizes and secrets vimentin from macrophages in the extracellular space, which in turn acts as DAMPs and activates Toll-like receptors 4 (TLR4)/NF-kB pathway in lung fibroblasts. These fibroblasts secrete pro-fibrotic cytokines TGF-*β*1, CTGF, and IL-8 (Baran et al., 2007).

The extrinsic risk factors for IPF include smoking, environmental exposures, and air pollution (Zaman and Lee, 2018). Cd, a heavy metal present in cigarette smoke, is found in high levels in the lungs of smokers (Ganguly et al., 2018). The phosphorylated forms of vimentin at Ser 38 and Ser 55 (P-Ser38 and P-Ser55 vim) resulted in Cd mediated peribronchial fibrosis in mice lungs. Our group has demonstrated that Cd-induced AKT and cdc2 activation increase phosphorylation of vimentin intermediate filaments Ser 38 (P-Ser38Vim). The P-Ser38Vim complexes with 14-3-3 for the release of YAP-1 for the translocation in nucleus triggering SMAD2/3 regulated transcription of pro-fibrotic genes in the fibroblasts around the airways (Li et al., 2017b). 14-3-3 is a conserved and regulatory phospho-binding protein with diverse roles in several signaling pathways (Pennington et al., 2018) and utilizes vimentin as a “sink” that sequester 14-3-3 away from binding partners (Tzivion et al., 2000; Pan et al., 2012; Sluchanko et al., 2017). 14-3-3 regulates autophagy through its interactions with Vimentin intermediate filaments. 14-3-3 forms autophagy-inhibitory Beclin1/14-3-3/vimentin intermediate filament complex for the pathogenesis of cancer (Wang et al., 2012). The dysregulation of autophagy is one of the pathogenic phenomena in IPF (Patel et al., 2012). The increased Vimentin intermediate filaments complexes with Beclin-1 to inhibit the clearance of CollagenI by autophagy in myofibroblasts. Increased ECM deposition and intemperate invasive capacity of myofibroblasts are hallmarks of IPF disease and are related to Vimentin intermediate filaments formation. Vimentin intermediate filaments are essential for invadopodia formation (Helfand et al., 2011). Moreover, we demonstrated that increased Vimentin intermediate filaments in myofibroblasts of fibrotic foci in the lungs of patients with IPF are related to the increased invasiveness of myofibroblasts and disease progression (Surolia et al., 2019). Overall, the ability of Vimentin intermediate filaments for interacting with other signaling molecules to form complexes regulates various pro-fibrotic pathways.

#### Role of Vimentin in COPD

Chronic inflammation leads to fixed narrowing of small airways (peribronchial fibrosis) and alveolar wall destruction (emphysema) in COPD. The chronic inflammation in COPD is characterized by increased numbers of alveolar macrophages, neutrophils, cytotoxic T-lymphocytes (O'Donnell et al., 2006). The increased activity of PAD2 in the macrophages (Makrygiannakis et al., 2008), likely contribute to the increased levels of citrullinated vimentin in the lungs and serum of COPD patients (Wood et al., 2011; Lugli et al., 2015; Nissen et al., 2019). However, patients with COPD have a specific form of vimentin which is believed to be a metalloproteases cleaved citrullinated form of vimentin (VICM) (Nissen et al., 2019). Neutrophil-specific protease membrane-type 6 matrix metalloproteinase (MT6- MMP) on neutrophil membrane utilizes vimentin as one of their substrate (Starr et al., 2012). Taken together, increased PADs and MMP activity on vimentin in the patients with COPD are the reason for increased levels of VICM. The downstream effects of VICM are not explored yet. In physiological conditions, the cleaved form of extracellular vimentin increases neutrophil and monocyte chemotaxis, generating “eat-me” signals that can potentially increase phagocytic removal of neutrophils to resolve inflammation. On the other hand, lungs from COPD patients are known to have compromised resolution of inflammation (Bozinovski et al., 2014). VICM may have differential responses on neutrophil and monocyte chemotaxis, phagocytosis, and the resolution of inflammation, which in part may be responsible for the frequent acute and chronic bacterial infections. For example, patients with COPD also have a higher prevalence of invasive pulmonary aspergillosis (IPA) (Bulpa et al., 2007; Samarakoon and Soubani, 2008) than those without COPD. Increased VICM levels can be plausible reason for the increased prevalence of *Aspergillus* species colonization in COPD patients. Non-TLR receptor, Dectin-1 has been explored in *Aspergillus* infections in the lungs (Lilly et al., 2012; Dutta et al., 2020). Dectin-1 contributes to respiratory burst, phagocytosis, and TNF-*α* production (Brown, 2006) and recognizes vimentin as a substrate (Thiagarajan et al., 2013). We think that prevalence of IPA in COPD may be associated with discrepancies in the binding of VICM to Dectin-1. These hypotheses are needed to be further tested.

#### Role of Vimentin in Autoimmunity Associated Interstitial Lung Diseases

Citrullinated vimentin was first recognized as an antigen for the autoimmunity in RA (Chen et al., 2015), among other citrullinated protein groups that cause anti-citrullinated peptide antibody production (ACPA). It is believed that the production of APCA initiates in the mucosa of the lungs before the onset of RA (Klareskog and Catrina, 2015; Zaccardelli et al., 2019). These observations indicate the possible connection of Cit-Vim antibodies to ILD development in RA patients (Chen et al., 2015; Reid and Guler, 2021). We infer that citrullinated vimentin may have a similar role as DAMP for developing RA-ILD based on the other research in IPF, but further investigation is necessary. One other form of post-translationally modified vimentin is recognized as a carbamylated-vimentin associated with cigarette smoking in patients with RA (Ospelt et al., 2017). Carbamylation is homocitrullination of proteins, where carbamylations are formed by the interaction of isocyanate (HNCO) with *α*-amino and ε-amino groups of proteins, among them, α-carbamylation, when *α*-amino groups of amino acids are involved, and ε-carbamylation, which is formed by the interaction of isocyanate with the ε-amino group of lysine (Jaisson et al., 2011). Although carbamylation is well-recognized in patients with RA, it is an APCA-independent process. The direct role of the carbamylated form of vimentin is not known in RA-ILD, IPF, and COPD. Interestingly, a recent study demonstrated that the global carbamylation of proteins by eosinophil peroxidase in the asthmatic airways participates in asthma exacerbations and altered inflammatory responses (Wang Z. et al., 2016). Recent research also has identified that elevated APCA levels were associated with asthma before the onset of RA disease (Zaccardelli et al., 2019). The direct role of citrullinated vimentin antigen and antibody in mucosal inflammation and asthma needs to be explored.

#### Role of Vimentin in Asthma

Asthmatic lungs have airway narrowing, and obstruction is intricately associated with EMT (Hackett, 2012). Inhaled environmental allergens promote EMT pathways *via* multiple mechanisms in the asthmatic airway. Hence, vimentin has been demonstrated in various airway epithelial cell types upon exposure to various allergens and other stimulants for EMT (Hackett, 2012).

### Lung Cancers

Vimentin is crucial for the EMT, metastasis, and invasion of mesenchymal cells (Satelli and Li, 2011; Usman et al., 2021). Hence, no wonder that most studies designated the significance of vimentin as a biomarker in cancers with clinical relevance in several types of cancers (Ben-Ze’ev and Raz, 1985; Hu et al., 2004; Jin et al., 2010; Li M. et al., 2010; Wei et al., 2008; Bogush et al., 2020) and the more in depth information can be found elsewhere (Satelli and Li, 2011; Zhao et al., 2013; Polioudaki et al., 2015; Mogre et al., 2022). In lung cancers, vimentin has been shown to be the target of various regulating factors that control expression or cause post-translational modification of vimentin. For example, increased activity of PARP-1 on the promoter of the vimentin gene increases the expression levels of vimentin (Rodriguez et al., 2013). In another study, cancer stem cell-derived exosomal miR-210-3p bind and inhibit fibroblast growth factor receptor-like 1 (FGFRL-1) to increase vimentin expression in lung cancer cells (Walls et al., 2020). Increased vimentin provides stability to FAK through VAV2-mediated Rac1 activation that increases the motility and invasiveness in non-small cell lung cancer (Havel et al., 2015). The decreased levels of post-translationally modified glycosylated-vimentin intermediate filaments are associated with the progression of adenocarcinoma (Rho et al., 2009). A recent study demonstrated that reduced glycosylation of vimentin increase the soluble form of vimentin [unit-length filaments (ULFs)] which is crucial for its self-assembly. (Tarbet et al., 2018). Expression of vimentin can also regulate the Slug signaling pathways for the pathogenesis of cancer (Vuoriluoto et al., 2011). These studies signify vimentin as an important driver and biomarker of EMT, increased migration, and metastasis in lung cancers.

In recent years, our understanding of the role of Vimentin intermediate filaments as a crucial player in the development of cancers by regulating non-EMT-dependent pathways has also evolved. Interaction of vimentin intermediate filaments with Beclin-1 inhibits autophagy enhancing tumorigenesis. The Akt1 mediated phosphorylated vimentin interact with 14-3-3 and complexes with Beclin1. The unavailability of Beclin1 for autophagosomal complex results in autophagy inhibition and cell survival in cancer (Wang et al., 2012). Similar work demonstrated that the interaction of Beclin1 with vimentin affects its USP14 mediated de-ubiquitination leading to abrogated degradation which provides an increased ability of cell migration in lung cancer (Cheng et al., 2019). These studies suggested that the formation of protein complexes by vimentin intermediate filaments can regulate new unconventional pro-tumor functions in the cell. Vimentin-associated intergenic cytoplasmic non-coding RNA inhibits Trim16 dependent polyubiquitination and degradation of vimentin intermediate filaments (Tian et al., 2020). These long-lived (unubiquitinated) vimentin intermediate filaments activate AKT-driven metastasis of adenocarcinoma (Tian et al., 2020). Overall, these few studies spotlight the regulatory role of vimentin intermediate filaments in unconventional ways, and more research is warranted to fully understand the role of vimentin in tumor metastasis.

## Vimentin as a Biomarker and as a Drug Target for the Lung Diseases

The aforementioned studies present evidence for the crucial role of Vimentin intermediate filaments in the development of lung diseases and prove that vimentin is a potential target for their treatment. Withaferin A and Ajoene are utilized as anti-vimentin strategies to treat *in vitro* and *in vivo* models of lung diseases. Withaferin A and Ajoene, both are plants compounds that inhibit the assembly of Vimentin intermediate filaments (Kaschula et al., 2019). Withaferin A, an alkaloid, is demonstrated to have anti-cancer effects review: Singh et al. (2021). Furthermore, Withaferin A decreased the invasiveness of IPF lung-derived 3D organoid models and mitigated lung fibrosis in the bleomycin mouse model (Surolia et al., 2019). Studies have shown that Withaferin A reduced inflammation in cellular models of cystic fibrosis (Maitra et al., 2009), and an ovalbumin mouse model of allergy and asthma (Zhao et al., 2019). Similarly, Ajoene which is a garlic compound has anti-cancer effects (Taylor et al., 2006; Wang Y. et al., 2016). Overall, these compounds or their derivatives have the potential as anti-vimentin targets for treating various lung diseases.

Although no direct anti-vimentin molecule is approved for the treatment of any lung disease, there are several clinical trials utilizing interventions that decrease the expression of vimentin. The value of amplified expression of vimentin is recognized as a prognostic marker is critical in non-small cell lung cancer (NSCLC) (Al-Saad et al., 2008; Dauphin et al., 2013; Ye et al., 2016; Teocharoen et al., 2021). The expression levels of vimentin were used as a prognostic indicator for the treatment efficacy for patients with NSCLC with erlotinib, erlotinib/bevacizumab (EB) or cisplatin/gemcitabine/bevacizumab (PGB) (Richardson et al., 2012; Villalobos et al., 2019). A phase I trial for FAK inhibitor drugs, namely VS-6063 and RO5126766, will use expression levels of vimentin as a biomarker in patients with NSCLC (NCT03875820).

There are new clinical trials for non-malignant lung diseases using vimentin as a biomarker and/or drug target (NCT03253146, NCT03584802). One of the studies is focused on the clinical value of vimentin and the mechanism of vimentin-mediated immune cell apoptosis during sepsis development. This trial will determine whether the vimentin can be a new target for sepsis diagnosis and treatment (NCT03253146). In an interventional clinical trial study, the autoantibodies to vimentin are being assessed as outcomes for the use of therapeutic plasma exchange, Rituximab, and IV IgG in the patients with severe acute exacerbation of IPF admitted in ICU (NCT03584802).

Simvastatin, a FDA-approved drugs have anti-vimentin effects (Trogden et al., 2018). Statins inhibits the isoprenylation of proteins which activates caspases. Vimentin is a well-known substrate for caspases (Byun et al., 2001). Simvastatin has anti-viral effects for Zika and HIV viruses (Esposito et al., 2016; Espano et al., 2019). Interestingly, vimentin has an important role in the invasion and replication of HIV and Zika viruses in host cells (Thomas et al., 1996; Cortese et al., 2017). As mentioned earlier, of vimentin has a plausible role in the host cell invasion during COVID infections (Ramos et al., 2020; Vora et al., 2021). Currently, the role of Ruxolitinib and Simvastatin therapy are being studied for the prevention and treatment of respiratory failure associated with COVID-19 (NCT04348695). A randomized double-blind placebo-controlled single-center trial has also demonstrated that Simvastatin significantly prolonged the time to first COPD exacerbation and reduced exacerbation rate (Schenk et al., 2021), NCT00680641).

Pritumumab (Glassy and Hagiwara, 2009; Babic et al., 2018) also known as CLNH11, CLN-IgG, and ACA-11, is the first anti-vimentin monoclonal antibody drug. It is a human IgG1 kappa antibody that binds to tumor cell ectodomain vimentin antigen for its anti-cancer effects. This drug is in clinical trial phase II, and showing beneficial effects against glioma (NCT04396717). Interestingly, testing of Pritumumab as a potential strategy for the anti-COVID 19 effects has been reported recently. Blocking the interaction of SARS-CoV2 spike proteins with surface vimentin co-receptor *via* the Pritumumab reduced the cell surface binding of the virus and cellular infection (Suprewicz et al., 2021).

### Concluding remarks

Cytoskeletal filament research areas are expanding to understand the emerging versatile role of intermediate filaments, specifically Vimentin intermediate filaments. Due to technological advancements in the last 30 years, Vimentin intermediate filaments have gained recognition not only as building blocks for the support, compartmentalization, and trafficking in the cells but also as signaling molecules. Blocking/cleavage of extracellular pathological forms, and overexpressing cell surface forms of vimentin by decoy peptides or antibodies can be one of the strategies to target vimentin in lung diseases such as autoimmune diseases, cancer, and infections. Nevertheless, it is challenging to develop explicit strategies to target the pathological forms of vimentin due to its pleiotropic functions and spatiotemporal distribution. A plethora of research studies demonstrate the beneficial effects of anti-vimentin strategies in the treatment of models of various lung diseases. We strongly believe that further in-depth studies are much needed, particularly to understand both the beneficial and deleterious effects of each of the forms of vimentin. These studies will evolve the understanding of the pleotropic effects of all different forms of vimentin, which will aid in the development of novel drug molecules to target vimentin with greater efficiency and without side effects.
